# Diagnostic value of STAF score in combination with D-dimer in cardioembolism

**DOI:** 10.1371/journal.pone.0204838

**Published:** 2018-10-01

**Authors:** Li-Bin Liu, Ya-Dong Guo, An-Ding Xu, Jie-Xi Zhong, Wen-Yan Zhuo

**Affiliations:** 1 Department of Neurology, Zhuhai Hospital of Jinan University, Zhuhai, China; 2 Departmentof Neurology, the First Affiliated Hospital of Jinan University, Guangzhou, China; Temple University, UNITED STATES

## Abstract

The aim of this study was to evaluate the diagnostic value of the Score for the Targeting of Atrial Fibrillation (STAF) in combination with the serum D-dimer (DD) levels in cardioembolism(CE).This study was a retrospective case-onlystudy, consecutively including patients with acute ischemic stroke. All patients were evaluated following the STAF scoring criteria and were classified according to the Trial of ORG 10172 in Acute Stroke Treatment (TOAST) etiology classification criteria. A total of 317 patients were enrolled, including 37 CE cases (11.67%). STAF ≥5 showed a sensitivity of 89% and a specificity of 91% for the diagnosis of CE, whereas DD >791.30 ng/mL had a sensitivity of 58% and a specificity of 78%. When the STAF was used in combination with the DD level, the sensitivity was 95%, and the specificity was 100%.STAF score is an excellent tool for the diagnosis of CE when combined with DD, and can facilitate the etiological classification of acute ischemic stroke.

## Introduction

Ischemic stroke can be divided into two types according to their etiology: cardioembolism (CE) and non-cardioembolic stroke. These two types of ischemic stroke have significantly different treatment regimens, especially in the choice of secondary prevention strategies. Therefore, early classification of acute ischemic stroke patients based on etiology facilitates the selection of appropriate secondary prevention strategies. However, precise etiological classification depends heavily on the perfect auxiliary examination. Moreover, CE has a lower diagnostic rate and a higher missed diagnosis rate in Chinese clinical practice than abroad. This disparity can largely be attributed to this dearth of targeted assessments. A reliable tool for the preliminary differential diagnosis of the etiological type is needed to guide further targeted testing and to determine the CE diagnosis, which will greatly improve the diagnostic rate of CE.

No preliminary differential diagnostic method has been broadly proven to be highly effective for the diagnosis of CE. Recent studies have shown that the Score for the Targeting of Atrial Fibrillation (STAF) can be used for initial screening for the presence of atrial fibrillation (AF) in patients with ischemic stroke[1], especially for occult paroxysmal atrial fibrillation [2], and has shown good result in the differential diagnosis of CE [3]. However, the effectiveness of this scoring system requires further confirmation [4,5]. The serum biochemical indicator D-dimer (DD) has also shown a certain value for CE diagnosis. Nevertheless, the cut-off value and diagnostic value are inconsistent in several studies[6–8]. Our previous study [9] showed that DD>791.30 ng/mL had a sensitivity of 58% and a specificity of 78% for the diagnosis of CE. Considering the limited diagnostic value of the single test available for the initial identification of CE at present, the use of a combination of tests may improve the diagnostic accuracy. The STAF score and serum DD levels are readily obtained from patients at an early time point during their hospital stay. To improve the diagnostic rate of CE, this study mainly employed receiver operating characteristic (ROC) curve analysis and a combination of tests to evaluate the diagnostic value of STAF score and its combination with DD for CE based on a database from our previous study [9].

## Subjects and methods

### Subjects

As described in detail previously [9], this retrospective, case-only study was approved by the medical ethics committee of the First Affiliated Hospital of Jinan University, Guangzhou. Written informed consent was not obtained and the patients’ records/information were anonymized and de-identified prior to analysis. We consecutively enrolled patients with acute ischemic stroke hospitalized in the Department of Neurology at the First Affiliated Hospital of Jinan University from January 2009 to December 2010. All patients had been discharged, and were identified the etiological subtypes before hospital discharge.

#### Inclusion criteria

Patients were chosen (1) whose cases conformed to the diagnostic criteria of the 2014 China cerebrovascular disease diagnosis and treatment guidelines for ischemic stroke; (2) whose time of stroke onset was not more than 7 days prior to inclusion; and (3) who had no history of ischemic stroke within the previous 6 months.

#### Exclusion criteria

Patients were excluded if they (1) were below 18 years of age; (2) had a diagnosis of hemorrhagic stroke and transient ischemic attack; (3) had systemic infections, autoimmune diseases, malignancies, serious liver or kidney diseases, heart failure, blood disorders, or other systemic thromboembolic pathologies; (4) had experienced a myocardial infarction, acute trauma, or surgery within the previous three months; (5) took long-term antithrombotic drugs (including anti-platelet drugs, anticoagulants, fibrinolytics) and statins lipid-lowering drugs, inflammation inhibitors, immunosuppressive agents or hormones); or (6) underwent thrombolytic therapy after admission.

## Methods

### Collection of general data

All of the patients’ data were collected from their medical records, including all of the demographic data (e.g., gender and age), the history of their present illness, National Institutes of Health Stroke Scale (NIHSS) score, all imaging information, including routine electrocardiogram (ECG), 24-hour Holter ECG, transthoracic echocardiography (including left atrial size), transesophageal echocardiography, ECG monitoring results and laboratory tests, including serum DD, blood lipids, blood glucose.

### Specimen detection method

To determine DD levels in the sera, 4 mL of fasting cubital venous blood was collected from each patient in the morning after the day they were admitted to the hospital, anticoagulated using sodium citrate, and then centrifuged at 3000 r/min for 10 minutes for the isolation of serum. DD levels were measured with a kit and immunoturbidimetry using an automatic biochemical detector which were both produced by Beijing Leadman Biochemistry Co., Ltd.

### STAF and ischemic stroke classification

The STAF score were obtained from all patients strictly following the scoring criteria [1]; the specific scoring items were shown in Table 1. Left atrial enlargement was defined as a left atrial diameter greater than 35 mm in transthoracic echocardiography in accordance with the criteria set by the Society of Diagnostic Medical Sonography in the United States [10]. Atherosclerotic stroke of the large arteries and occlusive stroke of the small arteries were absent according to the Trial of ORG 10172 in Acute Stroke Treatment (TOAST) etiology classification due to the lack of vascular etiology. All patients were classified according to the TOAST etiology classification criteria [11] and divided into five types: large-artery atherosclerosis (LAA), cardioembolism (CE), small-arteryocclusion (SAA), stroke of other determined etiology (SOE), and stroke of undeterminedetiology (SUE). The STAF score and serum DD levels were compared between these subtypes.

**Table 1:** Criteria and scoring for the STAF score.

Criteria		Points
Age, y	*>*62	2
	≤62	0
Baseline NIHSS score	≥8	1
	<8	0
Left atrial dilatation	Yes	2
	No	0
Vascular etiology	Yes	0
	No	3
Total		0 to 8

### Statistical analysis

The SPSS13.0 statistical software was used to analyze all of the data. First, all of the patients’ general data were analyzed; the chi-square test was used for the counted data, and an analysis of variance or a rank sum test was used to compare the measured data. The STAF score and DD levels had skewed distributions in each subtype and were expressed using median and quartiles. A two-sample or multi-sample rank sum test was used to compare differences between the groups. A ROC curve was used to determine the cut-off value and its sensitivity and specificity and to evaluate the authenticity and accuracy of the CE diagnosis. Combined test methods were used when CE was diagnosed based on two parameters.

## Results

### Baseline data

As described in detail from our previous study[9], there were a total of 317 patients studied (Table 2), including 202 cases of males (63.72%) and115 cases of females (36.28%), with an average age of 64.91 ± 12.33 years. The SOE cases were not included in the statistical analysis due to their small number. As shown in Table 2, no statistically significant difference was observed in the presence of diabetes, LDL-C levels, the proportion of smokers, or alcohol consumption among the various subtypes. The proportion of patients with hypertension was higher in the LAA group, and the proportion of females was higher in the CE group.

**Table 2:** Baseline data of various stroke subtypes.

Baseline parameters	LAA	CE	SAA	SOE	SUE	Total	*P* Value
Number	162 (51.10%)	37 (11.67%)	62 (19.56%)	3 (0.95%)	53 (16.72%)	317	0.000
Males	108 (66.67%)	13 (35.14%)	42 (67.74%)	2 (66.67%)	37 (69.81%)	202 (63.72%)	0.002
Age, years (mean±SD)	65.70±10.64	68.73±14.24	62.44±11.97	39.33±22.19	64.17±13.72	64.91±12.33	0.000
Hypertension	146 (90.12%)	22 (59.46%)	54 (87.10%)	1 (33.33%)	44 (83.02%)	267 (84.23%)	0.000
Diabetes	44 (27.16%)	6 (16.22%)	15 (24.19%)	0	13 (24.53%)	78 (24.61%)	0.581
LDL-C (mmol/L)	2.97±0.73	2.57±0.81	3.01±0.78	1.92±0.62	2.80±0.79	2.89±0.77	0.463
Smoking	64 (39.51%)	9 (24.32%)	26 (41.94%)	1 (33.33%)	19 (35.85%)	119 (37.54%)	0.308
Drinking	24 (14.81%)	2 (5.41%)	7 (11.29%)	0	8 (15.09%)	41 (12.93%)	0.438

### Specific results of classification

As described in detail from our previous study[9], all patients were classified into five subtypes, including 162 cases (51.10%) of LAA, 37 cases(11.67%) of CE, 62 cases (19.56%) of SAA, 3 cases (0.95%) of SOE, and 53 cases (16.72%) of SUE (Table 2). The 37 CE cases included 28 cases of patients with atrial fibrillation, 5 cases of patients with sick sinus syndrome, one patient who underwent mitral valve replacement surgery (without atrial fibrillation), one patient with a patent foramen ovale, one case of dilated cardiomyopathy and one case of mitral valve prolapse. Polycythemia vera, arteritis, arteriovenousmal formations and arteriovenous fistulae were observed in the three cases of SOE.

### STAF score for each subtype

Detailed results were shown in Table 3. The SOE group was not included in the statistical analysis because of the small number of cases. The SUE group included some cases with both LAA and CE and some cases in which the etiology could not be determined, so SUE was also excluded from the statistical analysis. The *χ*^*2*^ = 105.20 and *P* = 0.000 results from the multi-sample rank sum test indicated different STAF score among the subtypes; the highest score was observed with CE.

**Table 3:** STAF score for each subtype.

	AT	CE	SAA
STAF (median, quartiles)	2.9 (1.7)	6 (2)	2 (2)

### ROC curve analysis of the CE diagnosis based on the STAF

**Fig 1:**
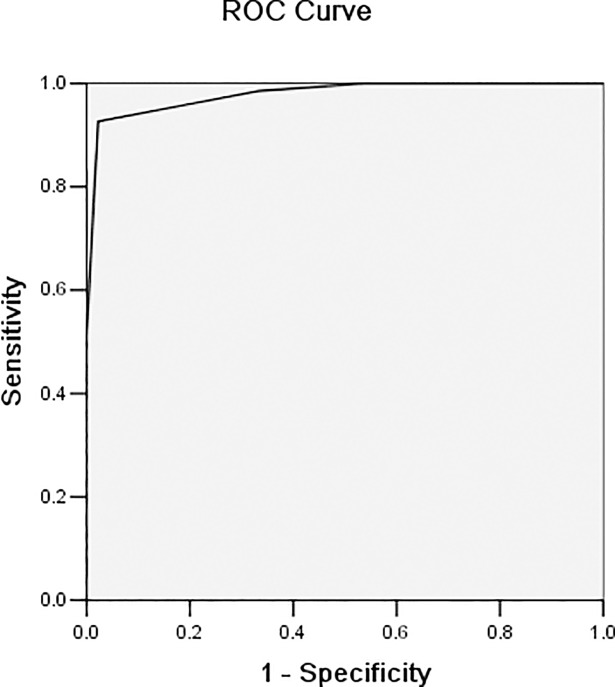
Predictive value of the STAF presented as ROC curve for the diagnosis of CE. The area under the ROC curve (AUC) was 0.929, which suggested good diagnostic value of the STAF for the diagnosis of CE.

To evaluate the diagnostic effect of the STAF for CE, its ROC curve was obtained using SPSS 13.0, and the cut-off value was determined. As shown in Fig 1 (Fig 1. Predictive value of the STAF presented as ROC curve for the diagnosis of CE), the area under the ROC curve (AUC) was 0.929 (generally considered to have diagnostic value when AUC> 0.5, low diagnostic value when AUC ≤ 0.7, moderate diagnostic value when 0.7 <AUC ≤ 0.9, and high diagnostic value when AUC> 0.9); thus, the result suggested high diagnostic value of the STAF for the diagnosis of CE. According to the cut-off value, STAF ≥ 5 has a sensitivity of 89%, specificity of 91%, positive predictive value of 90%, and negative predictive value of 93% for diagnosis of CE.

### STAF combined with DD for the diagnosis of CE

In our previous study [9], the cut-off value of DD used for the CE diagnosis was 791.30, which indicated that the CE type would be considered when DD was > 791.30 ng/mL; however, the sensitivity was only 58%; specificity, 78%; positive predictive value, 26%; and negative predictive value, 93%. As shown above, poor sensitivity was observed when CE was diagnosed based on STAF ≥ 5 or DD > 791.30 ng/mL alone, and the specificity of the latter measurement was also poor.However, STAF score could be used in combination with DD to improve the sensitivity and specificity, thereby increasing the diagnostic efficiency. If CE was diagnosed with STAF or DD greater than the cut-off value, then the two diagnostic methods could be used as parallel diagnostic tests to improve the test sensitivity (but reduced the specificity). The combined evaluation indicators shown in Table 4 were calculated as follows: sensitivity = 35/37 × 100% = 95%, specificity = 175/224 × 100% = 78%, positive predictive value = 35/84 × 100% = 42%, and negative predictive value = 175/177 × 100% = 99%.

**Table 4:** Evaluation of STAF and DD in combined diagnosis of CE (parallel diagnostic tests).

STAF and DD Value	Number of CE	Number of non-CE	Total
STAF≥5 or/and DD>791.30 ng/mL	35	49	84
STAF<5 and DD<791.30 ng/mL	2	175	177
Total	37	224	261

If CE wasonly diagnosed when both STAF and DD were greater than the cut-off values, then the two diagnostic methods could be used as serial diagnostic tests to improve the test specificity (but reduced the sensitivity). The combined evaluation indicators shown in Table 5 were calculated as follows: sensitivity = 20/37 × 100% = 54%, specificity = 224/224 × 100% = 100%, positive predictive value = 20/20 × 100% = 100%, and negative predictive value = 224/241 × 100% = 92%.

**Table 5:** Evaluation of STAF and DD in combined diagnosis of CE (series of diagnostic tests).

STAF and DD Value	Number of CE	Number of non-CE	Total
STAF≥5 and DD>791.30 ng/mL	20	0	20
STAF<5 or/and DD<791.30 ng/mL	17	224	241
Total	37	224	261

## Discussion

STAF is a scoring system [1] proposedby Suissa et al. in 2009 for screening for AF in ischemic stroke patients.Prediction of AF withSTAF ≥ 5 resulted in a sensitivity of89% and specificity of 88%, suggesting that such patients should be further subjected to multiple ECG examinations and repeated or extended 24-hour Holter ECG to evaluate the presence of paroxysmal or occult AF. The effectiveness of this test was confirmed in subsequent studies [2,5]. In 2016, Lin et al. [3] first found the excellent diagnostic value of STAF forthe classification of acute ischemic stroke patients based on etiology. STAF ≥ 5 had a sensitivity of 90%and specificity of 95% for predicting CEand could be used as a simple and accurate tool for the diagnosis of CE.Similarly, our study confirmed the good value of STAF for the differential diagnosis of CE, with a sensitivity of 89%, specificity of 91%, positive predictive value of 90%, and negative predictive value of 93% when STAF ≥ 5 was used alone to predict CE. The DD levelshowed a sensitivity of 58%, a specificity of 78%, apositive predictive value of 26%, and a negative predictive value of 93% when DD > 791.30 ng/mL was used alone to predict CE. To improve the accuracy, we utilized a test combination method to improve the combination sensitivity to 95%, with a combination specificity of 100% and a negative predictive value of the combination as high as 99%. The most valuable parameter of these evaluation indicators is the negative predictive value. For example, the negative predictive value was 93%for STAF ≥ 5, indicating that 93% of the ischemic stroke patients with STAF < 5 were definitely not the CE subtype.

The items involved in STAF score include patient data (age and NIHSS score at admission) and ultrasound radiography data (left atrial enlargement and vascular etiology), all of which are risk factors for AF [1]. This study suggests that STAF has a good diagnostic value for CE, which can be explained by the following aspects. First, the most common main risk factor for CE is AF [12], which is followed by myocardial infarction, heart failure, valvular heart disease, and sick sinus syndrome; these risk factors mostly occur in elderly patients [13–15]. In AF patients, approximately 70% have non-valvular AF, and 20% have valvular AF; the majority of the patients with non-valvular AF are elderly patients. Therefore, the elderly are more susceptible to CE. Second, compared to other types of ischemic stroke, CE has a more abrupt onset with more severe conditions and a higher NIHSS score [16,17]. Third, a number of recent studies have confirmed that left atrial enlargement is correlated with stroke and can increase the risk of stroke [18–20], especially the risk of CE [21].As an important etiological factor in stroke of undetermined etiology [22]and an independent risk factor for CE and the recurrence of cryptogenic stroke [23],left atrial enlargement is associated with the stroke severity [24].Additionally, left atrial enlargement may lead to local thrombosis [25]. Jaroch et al. found that a left atrial diameter greater than 51 mm was an independent risk factor for predicting thrombus in the left atrial appendage [26]. AF affects left atrial contraction and results in left atrial remodeling and then left atrial enlargement, leading to intra-atrial local blood stasis, which is most obvious in the left atrial appendage. Blood stasis results in raised local concentrations of coagulation factors, such as fibrinogen, leading to initiation of the coagulation process, induction of thrombosis, and possibly cerebral embolism [27–29]. Left atrial enlargement is also likely to increase the risk of AF [30]. In addition to AF, other organic heart lesions, such as myocardial infarction, atrial flutter, dilated cardiomyopathy, mitral valvular disease, valve replacement, atrial myxoma and patent foramen ovale, can cause left atrial remodeling and enlargement through affecting the afterload of the left atrium [31,32], and all of these conditions are risk factors for CE. In addition, the last item of STAF score (the absence of vascular etiology) refers to the exclusion of atherosclerotic stroke of the large arteries and occlusive stroke of the small arteries based on the TOAST etiological classification [1]. In summary, STAF score can be used to assist in the differential diagnosis of CE.

Multiple previous studies [6–8], including our previous study [9], have shown that the serum DD levels of patients with CE are significantly higher than those of patients with the other subtypes. The specific mechanisms of this finding have been previously explained and are not described here. DD is most likely a marker of CE, has a certain value for the diagnosis of CE, and may be used as an indicator for the exclusion of CE during early screening of ischemic stroke patients. At present, few studies have reported STAF score for the diagnosis of CE. This study has preliminarily demonstrated the diagnostic value of STAF. The combination of STAF and DD improved the diagnostic accuracy. However, further confirmation is needed with a large sample size. In this study, a serological indicator (DD), clinical data, and ultrasound radiography data (STAF score) were used in combination for the differential diagnosis of CE to identify patients who were suspected of or had a high risk of CE. This method provides guidance, especially for patients with cryptogenic or unexplained stroke, and offers strategic selection of targeted tests, such as transesophageal echocardiography, the transcranial Doppler (TCD)bubble test, long-term ECG monitoring, repeated 24-h Holter ECG, and cardiac magnetic resonance imaging (MRI), to improve the diagnostic rate of CE and expedite the most appropriate treatment and the initiation of secondary prevention programs.

## Conclusions

STAF score is a good tool for the diagnosis of CE that shows even better results when combined with DD and can facilitate the etiological classification of acute ischemic stroke.

## Supporting information

S1 File The raw data of AT patients. (DOCX)

S2 File The raw data of CE patients. (DOCX)

S3 File The raw data of SAA patients. (DOCX)
