# Supplementary material for: Diagnostic value of STAF score in combination with D-dimer in cardioembolism
Source: PLoS One. 2018 Oct 1;13(10):e0204838. doi: 10.1371/journal.pone.0204838 (PMC6166956; doi:10.1371/journal.pone.0204838)
Supplement: S1 File — (DOCX) [file pone.0204838.s001.docx]

| **Hospital number** | **STAF score** | **D-dimer(ng/mL)** |
| --- | --- | --- |
| 112770 | 4 | 2081.41 |
| 117399 | 2 | 259 |
| 120063 | 2 | 293.76 |
| 122052 | 4 | 892.38 |
| 123395 | 5 | 418.4 |
| 123534 | 2 | 946.56 |
| 126127 | 4 | 398.52 |
| 128734 | 2 | 240.45 |
| 130217 | 0 | 1533.06 |
| 137192 | 4 | 1702.86 |
| 139639 | 4 | 2051.73 |
| 141913 | 0 | 178.53 |
| 150728 | 4 | 745.29 |
| 151177 | 4 | 2372.97 |
| 158214 | 4 | 824.61 |
| 169876 | 2 | 393.12 |
| 169888 | 2 | 580.5 |
| 175331 | 4 | 330.70 |
| 176508 | 2 | 899.87 |
| 179736 | 2 | 2081.41 |
| 183834 | 4 | 294 |
| 187278 | 3 | 1443.57 |
| 194100 | 3 | 1218.56 |
| 194869 | 2 | 176.76 |
| 194453 | 2 | 281.6 |
| 198326 | 2 | 996.09 |
| 209037 | 2 | 2081.41 |
| 209928 | 2 | 807.50 |
| 215900 | 3 | 1151.94 |
| 218915 | 3 | 371.25 |
| 224000 | 2 | 357.82 |
| 224459 | 4 | 1657.31 |
| 225189 | 2 | 335.70 |
| 225750 | 0 | 221 |
| 226503 | 0 | 243 |
| 226686 | 2 | 1797.78 |
| 226912 | 0 | 137.73 |
| 227000 | 1 | 189.01 |
| 227145 | 0 | 127.02 |
| 227325 | 2 | 2741.93 |
| 227455 | 2 | 374 |
| 227789 | 2 | 266.77 |
| 227904 | 3 | 815.96 |
| 227938 | 3 | 763.2 |
| 228231 | 3 | 552.19 |
| 228466 | 0 | 328.67 |
| 228539 | 5 | 913.73 |
| 229136 | 4 | 470.2 |
| 229152 | 3 | 449.71 |
| 229557 | 3 | 97.71 |
| 229809 | 3 | 234 |
| 230015 | 3 | 594.60 |
| 230196 | 4 | 1146.13 |
| 230210 | 2 | 734.79 |
| 230345 | 2 | 1108.17 |
| 230623 | 2 | 1404 |
| 230856 | 3 | 732.82 |
| 230893 | 1 | 855.89 |
| 231071 | 2 | 459.57 |
| 231142 | 3 | 125.58 |
| 231254 | 1 | 286 |
| 231545 | 2 | 482.15 |
| 231551 | 3 | 1068.89 |
| 231677 | 3 | 571.55 |
| 232224 | 2 | 358.99 |
| 232360 | 0 | 270.57 |
| 232528 | 2 | 256.39 |
| 232711 | 2 | 497.87 |
| 232875 | 2 | 572.88 |
| 233366 | 2 | 428.89 |
| 234185 | 2 | 172.63 |
| 234478 | 3 | 1427.82 |
| 235061 | 4 | 146.12 |
| 235062 | 2 | 292.95 |
| 235205 | 3 | 712.06 |
| 235347 | 2 | 346.52 |
| 235385 | 3 | 456.53 |
| 235625 | 2 | 110.17 |
| 235660 | 2 | 221 |
| 235799 | 3 | 789.78 |
| 235825 | 1 | 1126.39 |
| 235890 | 0 | 172.6 |
| 235948 | 2 | 228.1 |
| 236095 | 3 | 698.55 |
| 236317 | 1 | 713.67 |
| 236536 | 0 | 150.08 |
| 236675 | 2 | 922.73 |
| 236940 | 2 | 708.39 |
| 293694 | 1 | 455.10 |
| 237200 | 1 | 185.43 |
| 237264 | 0 | 248.29 |
| 237388 | 1 | 943.3 |
| 237415 | 2 | 4037.02 |
| 237440 | 3 | 580.6 |
| 237617 | 3 | 3735.8 |
| 238216 | 4 | 401.02 |
| 238363 | 0 | 277.12 |
| 238472 | 2 | 212.8 |
| 238680 | 1 | 302.62 |
| 238691 | 2 | 566.46 |
| 238821 | 3 | 435.51 |
| 238972 | 2 | 293.61 |
| 239036 | 2 | 399.88 |
| 239604 | 3 | 552.83 |
| 240140 | 1 | 524.17 |
| 240183 | 1 | 335.73 |
| 240225 | 1 | 138 |
| 240257 | 2 | 626.16 |
| 240715 | 1 | 378.04 |
| 241506 | 1 | 139.69 |
| 241832 | 0 | 621.5 |
| 242106 | 4 | 501.79 |
| 242478 | 2 | 853.49 |
| 242508 | 0 | 169.83 |
| 242684 | 4 | 470.79 |
| 242970 | 2 | 323.58 |
| 243978 | 4 | 278.88 |
| 244278 | 0 | 385.09 |
| 244375 | 4 | 309.43 |
| 244387 | 3 | 796.55 |
| 244709 | 2 | 1088.81 |
| 244755 | 2 | 857.83 |
| 245728 | 0 | 2081.41 |
| 245935 | 2 | 1217.55 |
| 246093 | 2 | 691.15 |
| 246242 | 4 | 624.56 |
| 246870 | 4 | 726.25 |
| 246909 | 1 | 491.33 |
| 247585 | 2 | 224.22 |
| 247607 | 4 | 133.76 |
| 248152 | 0 | 196.16 |
| 248272 | 2 | 306.97 |
| 249203 | 5 | 669.92 |
| 249514 | 0 | 303.43 |
| 249893 | 3 | 203.71 |
| 250164 | 4 | 449.5 |
| 250268 | 2 | 81.82 |
| 250434 | 4 | 290.05 |
| 250513 | 4 | 1368.05 |
| 250651 | 2 | 483.92 |
| 250683 | 2 | 176.21 |
| 250950 | 4 | 660.46 |
| 251200 | 2 | 562.38 |
| 251369 | 2 | 487.87 |
| 251404 | 2 | 978.51 |
| 252124 | 2 | 128 |
| 252188 | 2 | 206 |
| 252336 | 3 | 364 |
| 252452 | 3 | 2081.41 |
| 252507 | 2 | 136 |
| 252949 | 2 | 162 |
| 253852 | 4 | 346 |
| 254376 | 3 | 413 |
| 254591 | 1 | 30 |
| 254725 | 0 | 145 |
| 254817 | 2 | 281 |
| 254958 | 3 | 703 |
| 255570 | 2 | 443.01 |
| 255730 | 5 | 188 |
| 255839 | 3 | 427 |
| 256558 | 2 | 550 |
| 256607 | 1 | 206 |
