# Supplementary material for: Diagnostic value of STAF score in combination with D-dimer in cardioembolism
Source: PLoS One. 2018 Oct 1;13(10):e0204838. doi: 10.1371/journal.pone.0204838 (PMC6166956; doi:10.1371/journal.pone.0204838)
Supplement: S2 File — (DOCX) [file pone.0204838.s002.docx]

| **Hospital number** | **STAF score** | **D-dimer(ng/mL)** |
| --- | --- | --- |
| 127377 | 5 | 2152.90 |
| 129849 | 5 | 1854.68 |
| 129969 | 7 | 291.13 |
| 139743 | 6 | 1327.9 |
| 168846 | 5 | 529.7 |
| 218215 | 6 | 902.96 |
| 220954 | 5 | 1097.88 |
| 223841 | 3 | 1975.32 |
| 226134 | 5 | 987.52 |
| 226686 | 6 | 1829.23 |
| 226768 | 6 | 4166.13 |
| 226858 | 5 | 158.89 |
| 229395 | 8 | 989.09 |
| 230183 | 7 | 131.75 |
| 230801 | 7 | 1693.04 |
| 232877 | 5 | 340.16 |
| 237636 | 5 | 225.29 |
| 239487 | 6 | 385.48 |
| 240822 | 5 | 1105.98 |
| 240833 | 5 | 1108.41 |
| 240957 | 7 | 801.14 |
| 242317 | 7 | 3293.20 |
| 242666 | 7 | 8282.67 |
| 242681 | 4 | 2005.21 |
| 243472 | 5 | 399.2 |
| 244691 | 7 | 1420.71 |
| 246402 | 4 | 545.25 |
| 247852 | 5 | 529.51 |
| 248037 | 5 | 973.10 |
| 248240 | 5 | 1961.46 |
| 249003 | 7 | 620.63 |
| 249586 | 5 | 1776.47 |
| 249705 | 6 | 792.81 |
| 251794 | 4 | 413.67 |
| 252606 | 7 | 161 |
| 253220 | 5 | 369 |
| 256926 | 5 | 226 |
