# Supplementary material for: Diagnostic value of STAF score in combination with D-dimer in cardioembolism
Source: PLoS One. 2018 Oct 1;13(10):e0204838. doi: 10.1371/journal.pone.0204838 (PMC6166956; doi:10.1371/journal.pone.0204838)
Supplement: S3 File — (DOCX) [file pone.0204838.s003.docx]

| **Hospital number** | **STAF score** | **D-dimer(ng/mL)** |
| --- | --- | --- |
| 132727 | 0 | 198 |
| 134616 | 2 | 692.21 |
| 146295 | 2 | 366.09 |
| 177620 | 2 | 1141.31 |
| 195581 | 0 | 137 |
| 196628 | 2 | 460.13 |
| 200630 | 4 | 229 |
| 202100 | 0 | 80.13 |
| 206683 | 0 | 103.91 |
| 220778 | 0 | 325.05 |
| 221073 | 2 | 305.79 |
| 226696 | 2 | 349.22 |
| 227145 | 4 | 1255.40 |
| 228283 | 0 | 161.11 |
| 228579 | 0 | 301.23 |
| 228879 | 2 | 291.13 |
| 229292 | 2 | 1363.02 |
| 229496 | 2 | 545.85 |
| 230214 | 0 | 248.22 |
| 230491 | 0 | 167 |
| 231480 | 2 | 502 |
| 232476 | 4 | 841.96 |
| 233067 | 0 | 358 |
| 233179 | 0 | 294.16 |
| 233182 | 2 | 118.36 |
| 233598 | 2 | 789.43 |
| 233832 | 0 | 4826.11 |
| 234284 | 0 | 129.57 |
| 234460 | 0 | 108.20 |
| 234579 | 2 | 451.45 |
| 234702 | 2 | 1331.51 |
| 235661 | 4 | 1219.18 |
| 235674 | 2 | 168.05 |
| 236590 | 2 | 329.85 |
| 266976 | 4 | 251.83 |
| 237237 | 0 | 84.71 |
| 237458 | 2 | 349.99 |
| 238941 | 0 | 272.9 |
| 239207 | 0 | 154.87 |
| 239509 | 2 | 361 |
| 239816 | 0 | 216.9 |
| 240328 | 2 | 295.19 |
| 240396 | 2 | 137.78 |
| 240488 | 0 | 137.25 |
| 240528 | 2 | 230.09 |
| 241906 | 2 | 168.56 |
| 242745 | 2 | 509.13 |
| 243260 | 2 | 866.5 |
| 244662 | 2 | 281.88 |
| 244854 | 0 | 249.58 |
| 245403 | 2 | 930.53 |
| 246601 | 2 | 425.88 |
| 249762 | 2 | 156.23 |
| 246862 | 2 | 252.24 |
| 250674 | 4 | 399.02 |
| 251531 | 0 | 193.7 |
| 252078 | 0 | 151.89 |
| 252826 | 0 | 193.87 |
| 252990 | 4 | 137 |
| 254039 | 2 | 79 |
| 255069 | 2 | 444 |
| 255392 | 2 | 322 |
